# Disordered Intestinal Microbial Communities During *Clostridioides difficile* Colonization and Subsequent Infection of Hepatic Cirrhosis Patients in a Tertiary Care Hospital in China

**DOI:** 10.3389/fcimb.2022.825189

**Published:** 2022-04-01

**Authors:** Yunbo Chen, Tao Lv, Dong Yan, Lisi Zheng, Beiwen Zheng, Jingxia Wang, Silan Gu, Lanjuan Li

**Affiliations:** ^1^ State Key Laboratory for Diagnosis and Treatment of Infectious Diseases, National Clinical Research Center for Infectious Diseases, Collaborative Innovation Center for Diagnosis and Treatment of Infectious Diseases, The First Affiliated Hospital, College of Medicine, Zhejiang University, Hangzhou, China; ^2^ Bacterial Research Platform, Jinan Microecological Biomedicine Shandong Laboratory, Jinan, China

**Keywords:** *Clostridioides difficile*, carriage, intestinal microbiota, 16S rDNA, hepatic cirrhosis

## Abstract

Patients with hepatic cirrhosis are more susceptible to *Clostridioides difficile* infection (CDI) and colonization with *Clostridioides difficile* (*C. difficile*). Asymptomatic *C. difficile* colonization is thought to predispose to subsequent CDI. However, the dynamic gut microbiota changes remain unclear. In this study, we used 16S rRNA gene sequencing to longitudinally monitor alterations in the intestinal microbiota of 22 hepatic cirrhosis patients with toxigenic *C. difficile* colonization at admission (pre-CDI) and developed CDI during hospitalization, subdivided into pre-CDI and CDI. 21 hospitalized cirrhotic patients without *C. difficile* colonization served as controls (HC). Compared with HC, pre-CDI and CDI samples had significantly decreased microbial richness and diversity, a significantly higher relative abundance of opportunistic pathogen *Enterococcus*, and a lower relative abundance of beneficial symbionts, such as *Faecalibacterium*, *Dorea*, and *Roseburia*. Three biomarkers showed high accuracy for distinguishing pre-CDI samples from HC with an area under the curve (AUC) up to 0.81. In conclusion, our study explored the changes of the gut microbiome before and after CDI. The gut microbial richness as well as diversity in CDI patients were notably reduced, relative to controls. Imbalance of the intestinal flora may be related to the risk for development of CDI. Identifying key members of the gut microbiota and illustrating their roles and mechanisms of action in CDI development are important avenues for future research.

## Introduction

*Clostridioides difficile* (*C. difficile*), an anaerobic spore-forming pathogen, is highly associated with intestinal infection and diarrhea following antibiotic treatment. *C. difficile* infection (CDI) is characterized by a spectrum of symptoms including abdominal pain, mild self-limited diarrhea, and can progress to toxic megacolon or even death in severe cases (Guh et al., 2020). The severity of CDI depends on the virulence of the strain, interactions with intestinal commensal microbiota, and the host defense and immune responses to intestinal epithelia damage induced by *C. difficile* (Abt et al., 2016). Patients with hepatic cirrhosis (HC) are usually hospitalized for abdominal infection, alimentary tract hemorrhage, and hepatic encephalopathy. These patients are more susceptible to CDI due to administration of anti-acid agent and antibiotics during hospitalization (Yan et al., 2017).

*C. difficile* colonization is common in the hospital, with a prevalence estimated at 3–26% among adult patients (Kampouri et al., 2021). Some research suggested that toxigentic *C. difficile* colonization decreased risk of CDI (Shim et al., 1998). However, numerous studies demonstrated that colonization is thought to contribute to CDI (Meltzer et al., 2019; Worley et al., 2021). By now, the risks for developing active CDI from *C. difficile* colonization are not well understood. However, it is important to identify those factors which could improve our understanding of the pathogenesis of this condition.

The intestinal microbiota provides resistance against CDI and disruption of the microbiota through e.g. antibiotic exposure may enhance susceptibility of individuals to the pathogenic effects of *C. difficile* (Herrera et al., 2021). Patients with CDI have a low diversity of intestinal microbiota and altered microbial composition (Gu et al., 2016). Previous research has demonstrated that CDI patients harbor a disrupted intestinal microbiota characterized by reduced levels of members of the *Lachnospiraceae* and *Ruminococcaceae* families, and *Prevotella* spp (Battaglioli et al., 2018; Han et al., 2019). Microbiota restoration through fecal microbiota transplantation (FMT), for example, re-introduces mechanisms of resistance that suppress *C. difficile* (Aroniadis et al., 2013). Peng et al. characterized the fecal microbiome of patients with CDI or asymptomatic *C. difficile* colonization and found lower microbial richness as well as diversity than in healthy control individuals, including a paucity of the Firmicutes and Bacteroidetes phyla and an overabundant Proteobacteria (Zhang et al., 2015). Another research also found that bacterial microbiota diversity decreased in both CDC and CDI patients (Crobach et al., 2020). However, these studies were limited to one time point, and therefore did not have a chance to capture the dynamic gut microbiota changes. Recently, based on longitudinal sampling, Berkell et al. showed that patients developing CDI exhibit significantly lower diversity and a distinct microbiota characterized by elevated levels of *Enterococcus* alongside reduced levels of *Ruminococcus*, *Blautia*, and *Prevotella* compared to non-CDI patients (Berkell et al., 2021). As the surveys have emphasized differences in gut microbial community structure between countries (Yatsunenko et al., 2012), there are no similar studies in China so far. In this prospective study, we investigated the intestinal microbiota of hospitalized patients during colonization with toxigenic *C. difficile* and subsequent CDI with the aim of identifying key members of the gut microbiota of CDI.

## Materials and Methods

### Study Design and Sample Collection

Hepatic cirrhosis patients who underwent *C. difficile* screening in the First Affiliated Hospital of Zhejiang University, a 5000-bed tertiary teaching hospital in Hangzhou, China, from May 2015 to October 2015, were enrolled in this study. We collected stool samples from patients within 48 h of admission and then at weekly intervals during hospitalization until either discharge or diagnosis with CDI. Once a patient develops diarrhea, stool specimens were immediately collected and tested for *C. difficile* culture and toxin genes.

Inclusion criteria included presence of cirrhosis but no hepatic carcinoma or other malignancies, ages 18-70 years, a Child–Pugh score of grade C, presence of toxigenic *C. difficile* colonization, CDI development during hospitalization, and hospitalization for more than one week. Patients with CDI diagnosis in the first 48 h of admission were excluded from the study as the infection may have been acquired prior to hospital admission.

All study participants provided the informed consent. Study protocols were permitted by the Ethical Committee of the First Affiliated Hospital, Zhejiang University School of Medicine.

### Patient Grouping

Of the 526 patients with cirrhosis, 22 met the inclusion criteria. The subjects involved 22 case patients who were confirmed as *C. difficile* colonization within 48 h of admission and then diagnosed as CDI during hospitalization and 21 hepatic cirrhosis controls (HC) from the same department who were neither infected nor colonized with *C. difficile* upon admission. A total of 65 fecal samples (44 from the case group and 21 from HC) were collected. Case patients were divided into two sub-groups: asymptomatic toxigenic *C. difficile* colonization (pre-CDI, n=22) and *C. difficile* infection (CDI, n=22). The asymptomatic carriers had a positive fecal culture for toxigenic *C. difficile* but did not exhibit diarrhea or other gastrointestinal disorders at the time of stool collection, within 48 h of admission (Loo et al., 2011). Patients with diarrhea (≥3 unformed stools in 24 h), whose fecal samples were found to be positive for *C. difficile* culture and toxin genes but negative for other pathogens, were diagnosed as having CDI (Bauer et al., 2009).

### Tracking of *C. difficile* in Fecal Samples

All stool samples were taken to the clinical microbiology laboratory at the First Affiliated Hospital of Zhejiang University in Hangzhou, China. Samples were aliquoted, one for *C. difficile* microbiology testing and the rest stored at -80°C within 24 hours of sample collection for subsequent assaying. Cultures of the stool samples for *C. difficile* was done using our previously described methods (Chen et al., 2014). Approximately 0.5 ml (0.5 g) of sample was diluted for 30 min in 0.5 ml 95% ethanol at room temperature (RT) for spore selection. We used cycloserine-cefoxitin-fructose agar supplemented containing 10% sheep blood for anaerobic isolation of *C. difficile.* Cultures were incubated for 48 h at 37°C, and the isolates confirmed as *C. difficile* by MALDI-TOF MS using the Microflex LT system (Bruker Daltonik GmbH, Bremen, Germany). As previously described, we used polymerase chain reaction (PCR) to assess toxin genes in *C. difficile* isolates, ie. *tcdA*, *tcdB* and *cdtA*/*cdtB* (Kato et al., 2001).

### Multilocus Sequence Typing (MLST) and Analysis

To investigate the population structure of isolates, we performed MLST on all toxigenic isolates with 7 housekeeping genes, ie. *adk*, *dxr*, *atpA*, *glyA*, *sodA*, *recA*, and *tpi*, as reported by Griffiths et al. (2010).The sequence type (ST) can be obtained by submitting the DNA sequence to the MLST database homepage (http://pubmlst.org/cdifficile/).

### Total DNA Extraction

We extracted the fecal genomic DNA from 0.2g of each sample using the QIAamp DNA Stool Mini Kit (Qiagen, Hilden, Germany) as instructed by the manufacturer and performed an additional homogenization step in a bead beater (FastPrep; Thermo Electron Corporation, Boston, MA, USA). A NanoDrop ND-1000 spectrophotometer (NanoDrop Technologies, Wilmington, DE, USA) was used to assess DNA quantity while DNA integrity as well as size was determined by electrophoresis on 1.2% agarose gels with ethidium bromide (0.5 mg/ml). The DNA was maintained at -20°C for subsequent analyses.

### 16S Ribosomal RNA Gene Sequencing

PCR amplification of V3 to v4 hypervariable regions of 16S rRNA gene were performed using the universal bacterial primers, 338F (5’-ACTCCTACGGGAGGCAGCAG-3’) and 806R (5’-GGACTACHVGGGTWTCTAAT-3’). We performed PCR analyses as: denaturation for 3 min at 95°C, 27 cycles for 30 s at 95°C, annealing for 30 s at 55°C, elongation for 45 s at 72°C, and 10 min at 72°C final extension. The resulting PCR products were extracted from a 2% agarose gel and further purified using a QIAquick Gel Extraction Kit (Qiagen, Hilden, Germany). Purified amplicons were pooled in equimolar concentrations and sequenced at paired ends with an Illumina MiSeq instrument (Illumina, San Diego, California, USA). All sequence data in this study was uploaded to the NCBI Sequence Read Archive (SRA) database (Accession Number: SRP159091).

### Sequencing Data Processing

Raw fastq files were demultiplexed, quality-filtered by Trimmomatic with default parameters and merged by FLASH. Operational taxonomic units (OTUs) with a similarity of 97% were clustered using UPARSE (version 7.1 http://drive5.com/uparse/) while UCHIME was used to identify and remove cheimeric sequences. Use the RDP classifier algorithm (http://rdp.cme.msu.edu/) for analyzing the classification of each 16S rRNA gene sequence targeting the 16S rRNA database with a 70% confidence threshold.

### Biodiversity and Microbial Community Analyses

We used mothur to calculate the ecological parameters for every fecal sample such as ACE and Chao 1 richness indices, Simpson diversity index and Shannon diversity indices, and the rarefaction curves which based on OTU counts. Variabilities in OTUs among pre-CDI, CDI, as well as control samples were evaluated by Principal coordinate analysis (PCoA). The Venn diagram to assess community overlaps was created by R. To analyze intestinal microbiota composition, comparative abundances of all determined microbial taxa were assessed by Linear discriminant analysis (LDA) effect size (LEfSe) (Segata et al., 2011) using pooled data from each group.

### Statistical Analysis

The SPSS 20.0 software and R packages (V3.6.1) were used for data analyses. We used Wilcoxon rank sum test to compare the crucial taxa between groups. Differences with a p-value of less than 0.05 were considered significant. For correlation analysis, Spearman’s rank test was performed. A random forest model was constructed for distinguishing between the HC and pre-CDI samples, and three genera as candidate biomarkers were selected on the basis of importance values (using the R package “randomForest”, ntree=500). We constructed the plotting receiver-operating characteristic (ROC) curves and calculating the area under the ROC curve (AUC) to assess the discriminate ability of the random forest model.

## Results

### Characteristics of the Participants

A total of 22 patients who were confirmed as *C. difficile* colonization at admission and then diagnosed as CDI during hospitalization (median age 56 years, interquartile range 47.8-63.5; 40.9% female) and 21 hepatic cirrhosis individuals (median age 52 years, interquartile range 44.5-67; 28.6% female) were enrolled. Differences in age, gender distribution, or body mass index between groups were not significant.

In the case group, patients suffered from colonization with *C. difficile* that produced toxin A and toxin B (A+B+) or only toxin B (A-B+). MLST was performed on the strains with positivity and a total of eight sequence types were identified. Basic information about the participants is displayed in **Table S1**.

### Sequence Reads and Ecological Diversity

**Figure 1:**
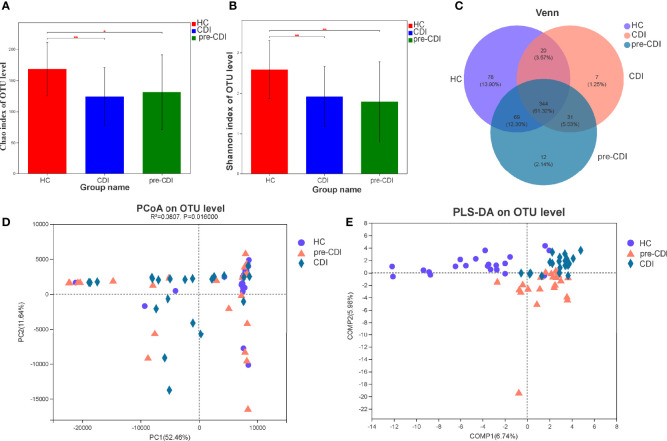
Structural comparison of fecal microbiota in the CDI, pre-CDI and HC groups. **(A)** The Chao1 and **(B)** Shannon indices were used to estimate abundance and diversity of the gut microbiota in patients (data are shown as the mean with SD). **(C)** A Venn diagram describing the overlap of OTUs in gut microbiome among the three groups. **(D)** PCoA of Euclidean distances at the OTU level. **(E)** PLS-DA score plot of gut microbiota among the three groups. *P < 0.05; **P < 0.01.

Sixty-five fecal samples (pre-CDI=22, CDI=22, HC=21) were collected from 43 subjects. A total of 1,960,725 sequences (30,165 reads per sample) can be used for downstream analyses after data manipulation and quality control with an average length of 445 bp. Compared with HCs, significant decrease in microbial abundance in samples from pre-CDI and CDI samples using the Chao1 index (p<0.05) (**Figure 1A**). According to the calculated comparison of the Shannon diversity index, microbial diversity was also lower in pre-CDI and CDI samples, relative to HC subjects (p<0.01) (**Figure 1B**). Rarefaction curves appeared no much fluctuation or growth along with the increasing of the size of our data (**Supplementary Figure S1**). Interestingly, differences in the indices of microbial diversity as well as richness between CDI and pre-CDI samples were insignificant (p>0.05; **Table 1**). A Venn diagram displaying the overlapping OTUs of the pooled samples falling into each sample type is shown in **Figure 1C**. The numbers of OTUs in pre-CDI, CDI, as well as hepatic cirrhosis control groups were 456, 402 and 511, respectively. Over 60% of the 561 OTUs were common to all samples, whereas 73.6% of OTUs were shared between the pre-CDI and HC groups (**Figure 1C**).

**Table 1:** Comparison of phylotypic coverage and diversity estimates of the 16S rRNA gene libraries with 97% similarity in pyrosequencing analysis.

Group ^a^	No. of reads	No. of OTUs^b^	Good’s(%)	Richness estimator ^c^		Diversity index ^c^	
				ACE	Chao1	Shannon	Simpson
CDI	663630	90.318 ± 36.297	99.92%	124.74 ± 39.049	123.96 ± 46.547	1.9161 ± 0.738	0.29752 ± 0.203
Pre-CDI	663630	102.14 ± 53.082	99.94%	138.1 ± 57.654	131.28 ± 59.868	1.7845 ± 0.992	0.37203 ± 0.297
HC	633465	136.38 ± 40.691	99.94%	170.31 ± 40.215	168.26 ± 42.957	2.5816 ± 0.717	0.18819 ± 0.183

^a^CDI, C. difficile infection; Pre-CDI, asymptomatic toxigenic C. difficile colonization; HC, hepatic cirrhosis controls.

^b^Data are expressed as the Mean ± Sd. The operational taxonomic units (OTUs) were defined at the 97% similarity level.

^c^No significant difference between CDI and Pre-CDI.

Principal coordinate analysis (PCoA) of Euclidean distances revealed that the overall microbial composition of pre-CDI and CDI subjects deviated from the HC controls (PERMANOVAR, R2 = 0.08, p = 0.02) (**Figure 1D**). Compared to the HC group, the CDI and pre-CDI samples showed greater heterogeneity in gut microbial communities. We also conducted partial least squares discriminant analysis (PLS-DA), a kind of supervised analysis suitable for high dimensional data. The bacterial communities in the CDI and pre-CDI samples clustered separately from the matched controls, which indicated that overall structure of the community differed markedly between the groups (**Figure 1E**).

**Figure 2:**
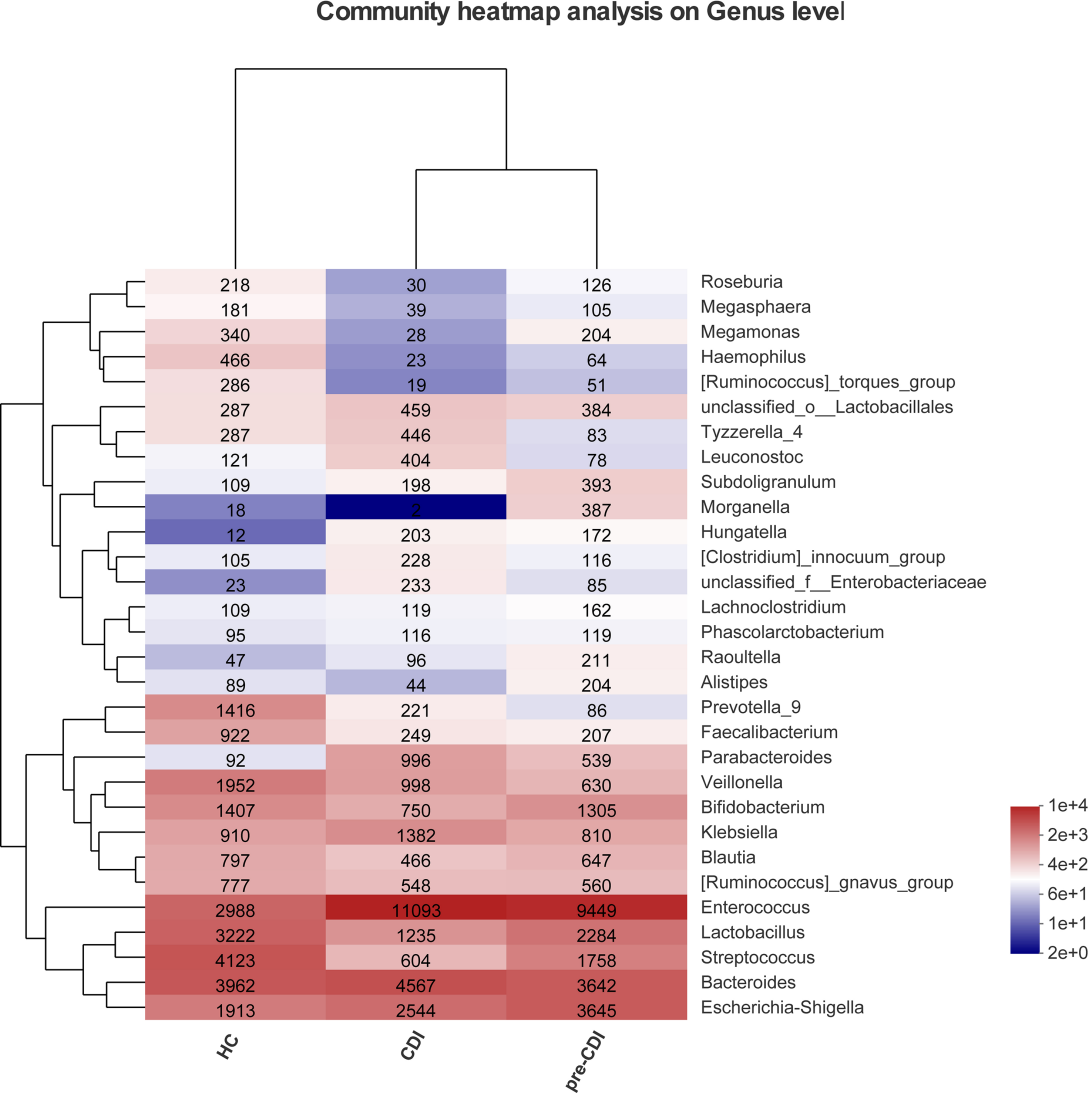
Heatmap representing the relative abundance of Top 30 genus-level in the CDI, pre-CDI and HC samples. The heatmap displays the abundant V3-V4 sequences of bacterial genera (rows) in three groups (columns). For each genus, the relative abundance is represented by specific values and a color gradient from dark blue (low abundance) to bright red (high abundance). The tree diagram displays the clustering in layers.

Sequence abundances by bacterial genera were compared across samples and the hierarchical clustering of top 30 dominant genera was depicted in the form of a heatmap (**Figure 2**). The heatmap showed that there were differences in abundance of the fecal microbial community in different groups. Compared with HC, pre-CDI samples had a higher relative abundance of opportunistic pathogens, such as *Enterococcu* and *Escherichia-Shigella*, and a lower relative abundance of beneficial symbionts.

### Distinctive Phylum- and Genus-Level Variations in Fecal Microbial Compositions Between Pre-CDI and HC Groups

**Figure 3:**
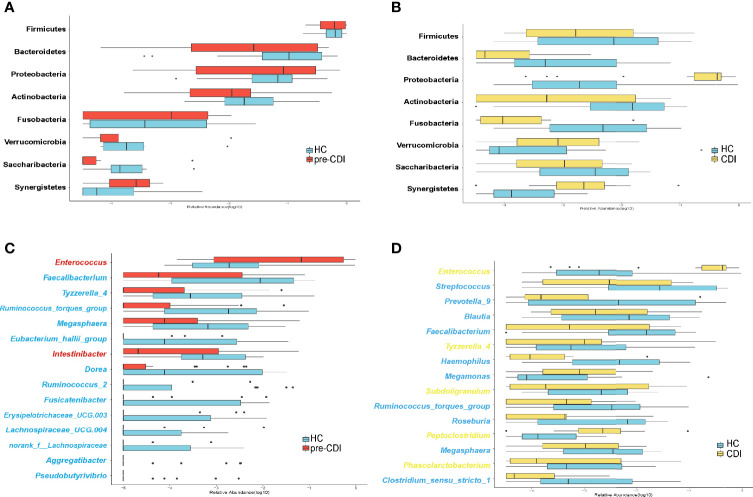
Microbiome alterations at the phylum and genus levels among the three groups. **(A)** The relative abundance of eight phyla between the pre-CDI (n=22) and HC samples (n=21). **(B)** The relative abundance of eight phyla between CDIs (n=22) and HC samples (n=21). **(C)** The relative abundance of 15 genera between the pre-CDI and HC samples. **(D)** The relative abundance of 15 genera between the CDI and HC subjects. The relative abundances of the phyla and genera are drawn on a log scale. Taxa enriched in HC patients are in blue text, and taxa overrepresented in pre-CDI and CDI samples are in red and yellow text, respectively.

Sequences were distributed among eight bacterial phyla, and the three most abundant and prevalent phyla were Firmicutes, Bacteroidetes, and Proteobacteria, which together harbored about 90% of sequences on average. However, Actinobacteria, Verrucomicrobia, Fusobacteria, Synegistetes, and Saccharibacteria accounted for 0.1% - 5% of sequences respectively. At the phylum level, differences among the three groups were insignificant (**Figures 3A, B**). At the genus level, we observed 15 bacterial taxa that displayed different abundance between pre-CDI and hepatic cirrhosis individuals. Two genera *Enterococcus* (p=0.027) and *Intestinibacter* (p=0.012) were enriched in pre-CDI subjects. Conversely, the abundance of several taxa *Faecalibacterium* (p=0.010), *[Ruminococcus]_torques_group* (p=0.003), *[Eubacterium]_hallii_group* (p=0.010), *Dorea* (p=0.011), and *Ruminococcus_2* (p=0.013) were significantly decreased in pre-CDI samples than hepatic cirrhosis controls (**Figure 3C**).

**Figure 4:**
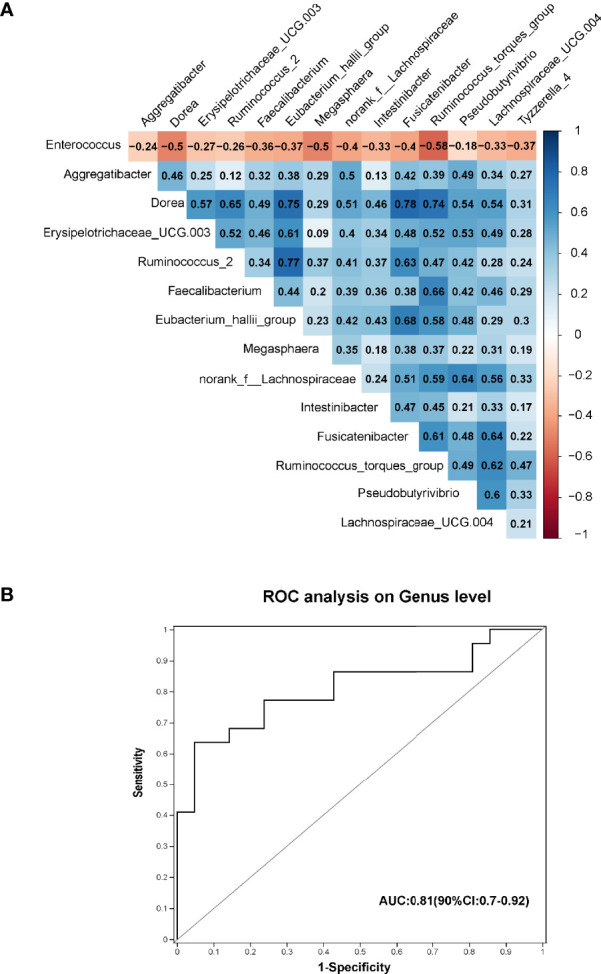
**(A)** Spearman correlations among the 15 significantly different genera in pre-CDIs (n=22) and hepatic cirrhosis controls (n=21). The microbial genera enriched in pre-CDIs (*Enterococcus*) were negatively correlated with control-enriched genera. **(B)** Prediction of the key genera in the gut microbiota of pre-CDI patients and controls with hepatic cirrhosis. Receiving operational curve analysis for *Intestinibacter*, *Allisonella and Haemophilus*, the area under the curve (AUC) was 0.81. Diagonal lines represent random classification (AUC=0.5).

Spearman correlation tests were conducted to assess the relationships between the 15 pre-CDI-related genera (**Figure 4A**). Remarkable positive correlations were observed in the pre-CDI-decreased genera, such as *Dorea* and *Fusicatenibacter* (ρ=0.78, p= 1.47E-14), *Ruminococcus_2* and *[Eubacterium]_hallii_group* (ρ=0.77, p= 2.58E-14), and *Faecalibacterium* and *Ruminococcus_torques_group* (ρ=0.66, p= 1.66E-09). *Enterococcus* spp. was abundant in pre-CDIs and showed a negative correlation with genera enriched in hepatic cirrhosis controls. To investigate the potential of using the gut microbiome to differentiate the HC and pre-CDI patients, we established a random forest model and selected three genera, *Intestinibacter*, *Allisonella and Haemophilus.* The AUC value of 0.81 (90%CI: 0.7-0.92) was obtained by evaluating the performance of the model using ROC analysis (**Figure 4B**).

### Variations in Intestinal Microbiota Between CDI and Healthy Control Groups

Compared to HC patients, the microbiota of CDI samples had a significantly higher abundance of the taxon *Enterococcus* (p=0.001), (*Pepto*)*clostridium* (p=0.002) and *Tyzzerella_4* (p=0.013). In contrast, *Streptococcus* (p=0.028), *Roseburia* (p=0.007), *Faecalibacterium* (p=0.010), *Megamonas* (p=0.029), Blautia (p=0.040), *Haemophilus* (p=0.007), *[Ruminococcus]_torques_group* (p=0.001) and *Clostridium_sensu_stricto_1* (p=0.012) associated with CDI patients had the most differentially depleted genera (**Figure 3D**).

### Longitudinal Alterations of the Microbial Compositions in Patients With Hospital-Acquired CDI

**Figure 5:**
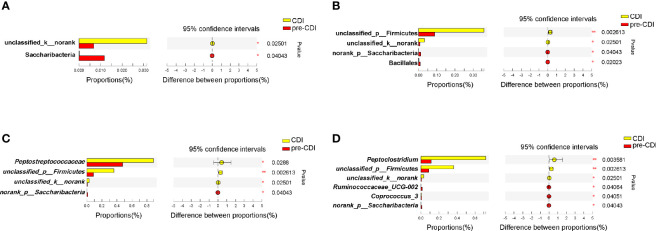
Taxonomic differences of fecal microbiota between the CDI and pre-CDI samples. Comparison of relative abundance proportions at the bacterial phylum **(A)**, order **(B)**, family **(C)** and genus **(D)** levels *P < 0.05; **P < 0.01.

As described above, the richness and diversity of microbiota did not differ significantly between the pre-CDI and CDI samples (p>0.05). In view of the dynamic gut microbiota changes, we analyzed the gut microbiota signature of them (**Figure 5**) and found the *Peptostreptococcaceae* (p=0.029) and (*Pepto*)*clostridium* (p=0.004) was markedly more abundant in CDI subjects. However, the genera *Ruminococcaceae_UCG-002* and *Coprococcus_3* were significantly higher in abundance in the pre-CDI samples (**Figure 5D**).

### Linear Discriminant Analysis (LDA) Effect Size (LEfSe) Reveals Remarkable Microbial Imbalance Among Patients

**Figure 6:**
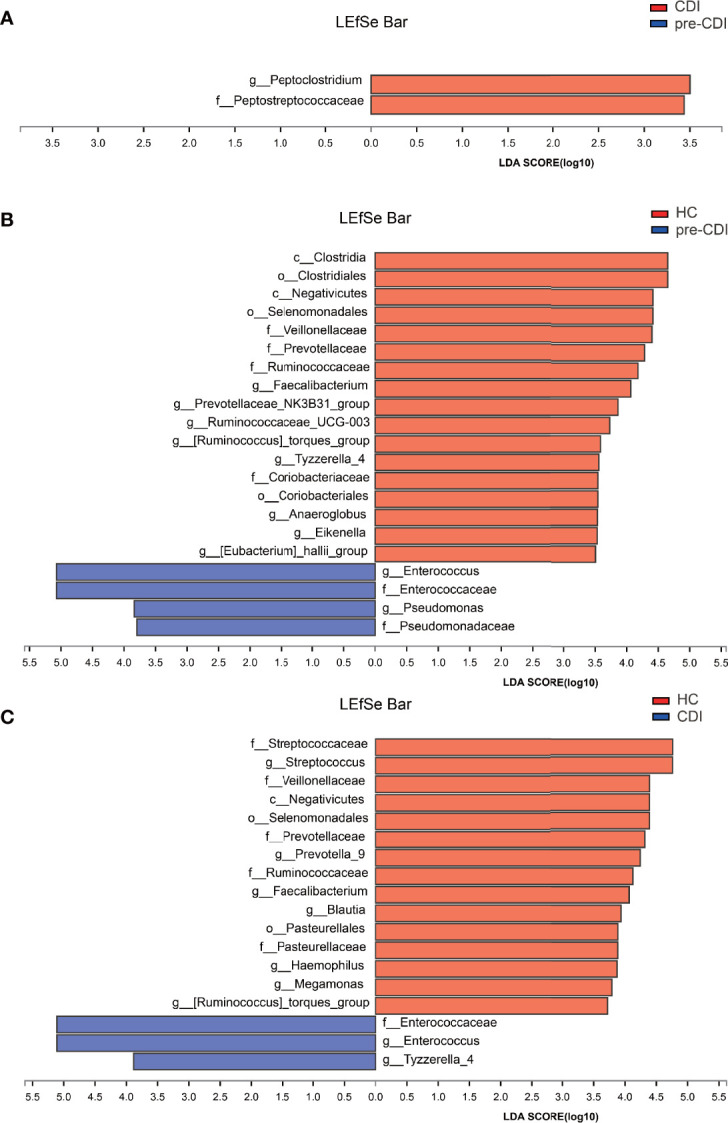
Linear discriminant analysis effect size (LEfSe) and Linear discriminant analysis (LDA) based on operational taxonomic units characterize microbiomes in the three groups. Only taxa that meet the LDA significance threshold >3 are displayed. The enrichment degree is proportional to the LDA score. **(A)** LDA scores indicate significant differences in the microbiota between the pre-CDI and CDI samples. **(B)** LDA scores indicate significant differences in the microbiota between the pre-CDI patients and HC. **(C)** LDA scores indicate significant differences in the microbiota between the CDI patients and HC.

We used the LEfSe software program to identify putative key microbial biomarkers of high risk of CDI development. Compared with pre-CDI samples, the gut microbiome of the CDI samples was dominated by (*Pepto*)*clostridium* and *Peptostreptococcaceae*, of which *C. difficile* is a member (**Figure 6A**).

Compared with HC individuals, the microbiome of the pre-CDI samples was dominated by *Enterococcus* and *Pseudomonas*, whereas the microbiome of HC had a dominance of *Faecalibacterium*, *Ruminococcaceae_UCG-003*, *Anaeroglobus*, *[Eubacterium]_hallii_group* and other beneficial bacteria. Many of these genera harbor short-chain fatty acid (SCFA) producers (**Figure 6B**). Differential abundance analysis also revealed several taxa with a signifcantly higher and lower abundance, respectively, between the CDI and HC groups (**Figure 6C**). Underrepresented taxa included *Faecalibacterium*, *Blautia*, *Streptococcus*, *Megamonas*, *[Ruminococcus]_torques_group*, *Ruminococcaceae* and *Streptococcaceae*. However, taxa such as *Enterococcaceae*, *Enterococcus* and *Tyzzerella* accounted for notably higher proportions in the samples from CDI subjects.

## Discussion

The complex gastrointestinal microbiota has a vital role in human health and disease, including nutrient metabolism and development of immune system, as well as providing protection against infection (Sorbara et al., 2020). Changes in the concentration of microbial and host metabolites create a favorable environment for *C. difficile* sporulation, germination, and toxin production that contribute to CDI (Buffie et al., 2014; Theriot et al., 2014; Fishbein et al., 2022).

The role of asymptomatic *C. difficile* carriers as a source of transmission and infection is increasingly recognized, especially in hospitalized patients (Blixt et al., 2017). Our previous research found that cirrhotic patients with toxigenic *C. difficile* colonization highly predisposed to CDI upon admission or during hospitalization (Yan et al., 2017). Risk of infection is nearly six times higher in *C. difficile* colonized patients than in non-colonized patients (Mollard et al., 2019). In contrast, risk reduction was found upon colonization with either nontoxigenic or toxigenic strains (Nagaro et al., 2013; Longtin et al., 2016). It has reported that asymptomatic *C. difficile*-colonized individuals may be sheltered from developing infection as they may induce a humoral immunological response to *C. difficile* toxins (Shim et al., 1998). The processes from asymptomatic colonization to infection are related to various elements, including the host native microbiota, bacterins, toxin A receptors as well as immunological aspects, and pathogen-associated variables (Schäffler and Breitrück, 2018). After receipt of antibiotics, the intestinal commensal bacterial component may become depleted, resulting in the destruction of colonization resistance and facilitating the progression of CDI (Lesniak et al., 2021). Previous studies have depicted the gut microbial composition of CDI patients and comparable findings concerning markedly reduced diversity and richness are observed driven primarily by a lack of Bacteroidetes and Firmicutes phyla (Antharam et al., 2013; Gu et al., 2016).

Although there is a strong association between gut microbiota and CDI susceptibility, the influence of toxicogenic *C. difficile* colonization on the microbiota and progression from asymptomatic colonization to infection in hepatic cirrhosis patients has not been examined prospectively in detail. The objective of this research was to assess the relationship between gut microbiome composition and the colonization and subsequent infection of *C. difficile* in hepatic cirrhosis patients. In our study, dynamic follow-up was given to 22 hepatic cirrhosis patients who had a toxigenic *C. difficile* carrying status and were presenting CDI during the hospital stay.

Consistent with previous studies (Antharam et al., 2013; Zhang et al., 2015), 16S rRNA genomic analysis demonstrated significant differences in microbial community structure between the pre-CDI, CDI and HC samples, as characterized by reduced microbial diversity and richness. Notably, a comparative paucity of SCFA-producing *[Ruminococcus]_torques_group*, *Dorea*, and *Roseburia* (from the *Lachnospiraceae* family), and *Faecalibacterium* and *Ruminococcus* (from the *Ruminococcaceae* family) were observed in *C. difficile* carriers. However, there were no marked variations in microbial richness and diversity between CDI and pre-CDI samples. It is clear that a sufficient density and diversity of gut microbiota is important to protect against *C. difficile* infection. *C. difficile*-colonized individuals may not directly progress to symptomatic CDI, which is dependent on the intestinal microenvironment, host immunity, and pathogen-related factors.

Gut microbiota of pre-CDI samples in this study was associated by low bacterial diversity, increased abundance of *Enterococcus*, and lower abundance of the butyrate-producing anaerobic bacteria, *Lachnospiraceae* and *Ruminococcaceae*. These findings confirm previous study reporting a strong correlation between *Lachnospiraceae* family members and colonization resistance to CDI, and several of these members are butyrate-producing bacteria (Mahnic et al., 2020). However, in Crobach et al’s report, they found that microbiota of CDI patients was characterized by a lower abundance of genera belonging to the *Ruminococcaceae* family (Crobach et al., 2020). The inconsistency may be due to the differences in patient selection, as we collected consecutive samples from the same patient in this study. Microbiota–produced butyric acid and other short-chain fatty acids play an important function in resisting pathogenic bacteria, suppressing inflammation, and enhancing intestinal defensive barriers through increased levels of antimicrobial peptide and production of mucin (de Vos et al., 2022). For asymptomatic carriers, disruption of the indigenous microbiome and consumption of butyrate-producing bacteria in the intestine may contribute to increase susceptibility to CDI. Meanwhile, levels of the *Faecalibacterium* genus were significantly reduced in pre-CDI samples*. Faecalibacterium* spp. is a key supplier of butyrate and shown to have an anti-inflammatory and protective effect in inflammatory bowel disease models of colitis (Sokol et al., 2010; Flanagan et al., 2011).

In addition to butyrate-producing bacteria, reduced levels of secondary bile salt-producing *Clostridium* clusters were observed in the pre-CDI and CDI samples. Certain bile acid 7-dehydroxylating intestinal bacteria within the *Eubacterium* and *Clostridium* genera, in particular *Clostridium scindens*, is proven to strongly resist *C. difficile* expansion in the gut microbiome of antibiotic-treated mice in a secondary bile acid-dependent fashion (Buffie et al., 2014). In contrast, significant increases in *Enterococcus* were found in *C. difficile* colonization and CDI samples than HC patients. Berkell et al. found that patients developing CDI already exhibit significantly lower diversity before antibiotic treatment and a distinct microbiota enriched in *Enterococcus* compared to non-CDI patients (Berkell et al., 2021). This observation further supports previous studies indicating that *Enterococcus* is more prevalent and more abundant in patients with *C. difficile* colonization and infection (Ozaki et al., 2004; Gu et al., 2016). Members of the *Enterococcaceae* family are commonly multidrug resistant opportunistic pathogens that may overgrow as a result of antimicrobial use (Lupp et al., 2007). On the other hand, the probiotic potential of *Enterococcus* has been acknowledged and is shown to have inhibitory activities against *C. difficile* (Romyasamit et al., 2020).

Studies indicate that patients with hepatic cirrhosis disease are more likely to contract *C. difficile* colonization and CDI (Eyre et al., 2013; Alasmari et al., 2014; Mantri et al., 2021). If asymptomatic carriers are common among hospitalization patients and put them at elevated risk of going from colonization to clinical infection, then prevention of progression can reduce the rate of hospital-onset CDI. Routine screening for *C. difficile* colitis in patients with cirrhosis is highly associated with improved healthcare outcomes and reduced health care service use (Saab et al., 2015). Thus, it is reasonable to raise attention to the toxigenic *C. difficile* colonization of cirrhotic patients in clinical practice.

This study had a small sample size and could not exclude a potential role for non-CDI factors in the gut microbiota. However, the findings of this study provided a first step in exploring how gut microbiota may act as a risk element for CDI. These data can inform disease surveillance and use of specific probiotics to prevent and treat CDI. Our results may help to identify individuals at a higher risk for *C. difficile* infection. Nevertheless, this cannot yet be generalized to other patient groups with different demographics characteristics, suggesting a need for bigger cohort research that includes patients with diverse demographics. 

In conclusion, our study prospectively explored commensal microbiota associated with hospital-associated pre-CDI and CDI. The gut microbial richness as well as diversity were markedly reduced in asymptomatic toxigenic *C. difficile* carriers and CDI samples, relative to the controls. Imbalance of the intestinal flora, particularly depletion of certain SCFA-producing bacteria, may contribute to the development of CDI or to disease susceptibility. Identifying key members of the gut microbiota and illustrating their roles and mechanisms of action in CDI development are important avenues for future research.

## Data Availability Statement

The datasets presented in this study can be found in online repositories. The names of the repository/repositories and accession number(s) can be found below: https://www.ncbi.nlm.nih.gov/, SRP159091.

## Ethics Statement

The studies involving human participants were reviewed and approved by the Ethical Committee of the First Affiliated Hospital, Zhejiang University School of Medicine. The patients/participants provided their written informed consent to participate in this study.

## Author Contributions

Study concept and design by SG and LL. Analysis and interpretation of data by YC, TL, and DY. Drafting of the manuscript by YC and SG. Critical revision of the manuscript for important intellectual content by BZ. Statistical analysis by LZ and JW. All authors contributed to the article and approved the submitted version.

## Funding

This study was sponsored by grants from the National Natural Science Foundation of China (No. 81800457 and 82073609) and Research Project of Jinan Microecological Biomedicine Shandong Laboratory (JNL-2022001A and 2022037C).

## Conflict of Interest

The authors declare that the research was conducted in the absence of any commercial or financial relationships that could be construed as a potential conflict of interest.

## Publisher’s Note

All claims expressed in this article are solely those of the authors and do not necessarily represent those of their affiliated organizations, or those of the publisher, the editors and the reviewers. Any product that may be evaluated in this article, or claim that may be made by its manufacturer, is not guaranteed or endorsed by the publisher.
