# Supplementary figures and images for: Disordered Intestinal Microbial Communities During Clostridioides difficile Colonization and Subsequent Infection of Hepatic Cirrhosis Patients in a Tertiary Care Hospital in China
Source: Front Cell Infect Microbiol. 2022 Apr 1;12:825189. doi: 10.3389/fcimb.2022.825189 (PMC9010725; doi:10.3389/fcimb.2022.825189)

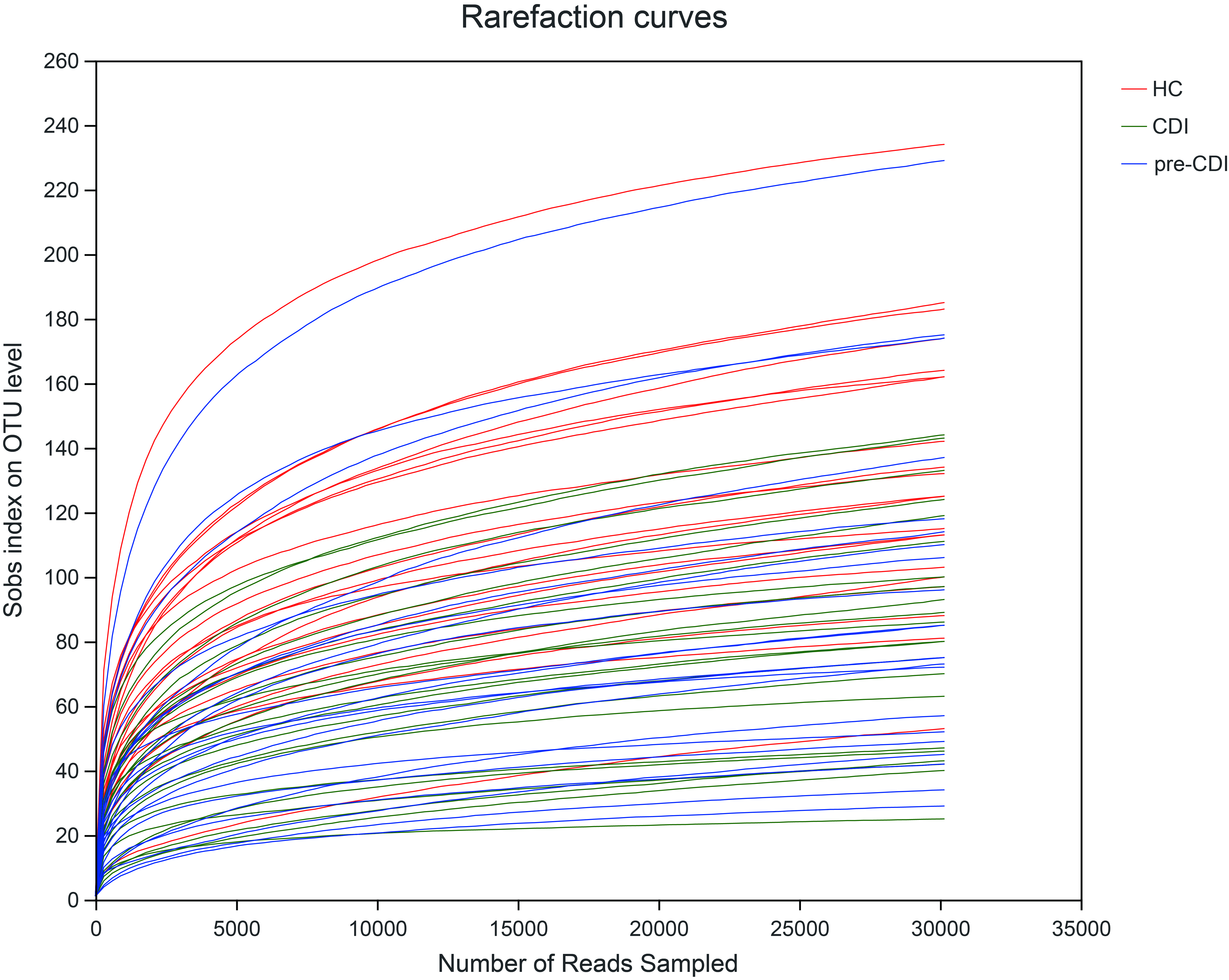

Supplement: Supplementary file 1 [file Image_1.jpeg]
